# Cognitive effects of genetic variation in monoamine neurotransmitter systems: A population-based study of *COMT*, *MAOA*, and *5HTTLPR*

**DOI:** 10.1002/ajmg.b.31150

**Published:** 2010-12-16

**Authors:** Jennifer H Barnett, Ke Xu, Jon Heron, David Goldman, Peter B Jones

**Affiliations:** 1 Department of Psychiatry, University of CambridgeCambridge, UK; 2 Cambridge Cognition LtdCambridge, UK; 3 Department of Psychiatry, Yale UniversityNew Haven, Connecticut; 4 Department of Social Medicine, Bristol UniversityBristol, UK; 5 Laboratory of Neurogenetics, National Institute on Alcohol Abuse and AlcoholismRockville, Maryland; 6 MRC/Wellcome Trust Behavioural & Clinical Neurosciences Institute (BCNI)Cambridge, UK

**Keywords:** genetics, cognition, serotonin, dopamine

## Abstract

Individual differences in cognitive function are highly heritable and most likely driven by multiple genes of small effect. Well-characterized common functional polymorphisms in the genes *MAOA*, *COMT*, and *5HTTLP*R each have predictable effects on the availability of the monoamine neurotransmitters dopamine, noradrenaline, and serotonin. We hypothesized that *5HTTLPR* genotype would show little association with prefrontal cognitive performance, but that *COMT* and *MAOA* would have interacting effects on cognition through their shared influence on prefrontal catecholamine availability. We assessed the individual and epistatic effects of functional polymorphisms in *COMT*, *MAOA*, and *5HTTLPR* on children's prefrontal cognitive function in nearly 6,000 children from the population-based Avon Longitudinal Study of Parents and Children (ALSPAC). Neither *MAOA* nor *5HTTLPR* polymorphisms showed significant effects on cognitive function. In boys but not girls, there was a modest but statistically significant interaction between *MAOA* and *COMT* genotypes such that increased prefrontal catecholamine availability was associated with better working memory. These results suggest that assessment of multiple genes within functionally related systems may improve our understanding of the genetic basis of cognition. © 2010 Wiley-Liss, Inc.

## INTRODUCTION

Experimental manipulation of human and primate neurotransmission has provided insight into the distinct roles that the monoamine neurotransmitters serotonin (5-HT), dopamine (DA), and noradrenaline (NA) play in prefrontal cognition [Clarke et al., 2004; Chamberlain et al., 2006; Ramos and Arnsten, 2007[. For example, while prefrontal DA is central to working memory [Brozoski et al., 1979[, experimental lowering of serotonin has little effect on prefrontally mediated tasks, but does impair learning [Park et al., 1994; Clark et al., 2005; Talbot et al., 2006[. Differentiating the cognitive effects of NA from its precursor, DA, can be difficult, but drugs which specifically mimic NA actions at a2 adrenergic receptors restore prefrontal cortex (PFC) function in catecholamine-depleted monkeys and rats [Arnsten and Goldman-Rakic, 1985; Carlson et al., 1992[ and improve executive functions in patients [Mair and McEntee, 1986; Fields et al., 1988; Taylor and Russo, 2001[ and healthy volunteers [Jakala et al., 1999[.

Two major routes for the deactivation of monoamine neurotransmitters are reuptake by transporter molecules in the presynaptic cell membrane, and enzymatic degradation within the neuron or synapse. Both serotonin and NA transporters are abundant in PFC [Mantere et al., 2002; Miner et al., 2003[ but in primates the DA transporter is located primarily extrasynaptically [Lewis et al., 2001[, resulting in a more prominent role for enzymatic degradation of synaptic DA. It is estimated that catechol-*O*-methyltransferase (COMT), one such enzyme [Karoum et al., 1994; Gogos et al., 1998[, may be responsible for half of prefrontal DA decline [Yavich et al., 2007[. In contrast, COMT appears to have relatively little effect on NA in frontal cortex: *COMT* knockout mice show normal prefrontal NA levels and administration of the COMT-inhibitor tolcapone increases the release of DA, but not NA, in rat PFC [Gogos et al., 1998; Tunbridge et al., 2004[. A second set of enzymes, the monoamine oxidases, catalyze the oxidation of monoamine neurotransmitters, including serotonin, DA, and NA from their location in the mitochondrial outer membrane [Green and Youdim, 1975[. The A isoform (MAOA) is more abundant in catecholaminergic neurons, while MAOB is more abundant in serotonergic neurons [Levitt et al., 1982; Westlund et al., 1988; Willoughby et al., 1988; Saura et al., 1992[.

Individual differences in cognitive function are highly heritable [Devlin et al., 1997[, and this may be driven partly by genetic variants, which directly affect prefrontal neurotransmitter availability. Functional polymorphisms in *COMT*, *MAOA*, and the serotonin transporter-linked polymorphic region (*5HTTLPR*) affect the level and/or activity of their products in defined manners, summarized in Table I. Cognitive effects of these functional polymorphisms have been studied to varying extents. Egan et al. [2001[ reported that the *COMT* Val allele was associated with worse performance on a measure of executive function, the Wisconsin Card Sort Test (WCST). While meta-analysis of similar studies shows little consistent cognitive effect [Barnett et al., 2008[, consistent differences are seen in prefrontal activation during working memory tasks, with greater activation among Val allele carriers [Egan et al., 2001; Mier et al., 2010[. In contrast, relatively little is known about the effects of the *MAOA* and *5HTTLPR* polymorphisms on cognition. *MAOA* genotype has been reported to significantly affect IQ among healthy Chinese women [Yu et al., 2005[ and children with autism [Yirmiya et al., 2002; Cohen et al., 2003[ but with conflicting directions of effect. Reports regarding cognitive effects of the *5HTTLPR* polymorphism have been predominantly negative for standard neuropsychological measures such as IQ, response inhibition, and sustained attention [Fallgatter et al., 1999; Clark et al., 2005; Payton et al., 2005; Roiser et al., 2007; Strobel et al., 2007; da Rocha et al., 2008; Borg et al., 2009[, though there are now multiple reports of associations with risk-taking, decision-making, and attentional biases [Beevers et al., 2007; Homberg et al., 2008; da Rocha et al., 2008; Crisan et al., 2009; Firk and Markus, 2009; Fox et al., 2009; Roiser et al., 2009[.

**TABLE I:** Genetic Polymorphisms Examined in the Avon Longitudinal Study of Parents and Children (ALSPAC)

Gene	Effects on 5-HT	Effects on NA	Effects on DA	Polymorphism	Functional effects	Predicted effects on prefrontal cognition
Monoamine oxidase A; (MAOA)	Yes	Yes	Yes	30-bp VNTR in promoter	3-repeat allele results in approximately fivefold lower transcription than 3.5- or 4-repeat alleles [Sabol et al., 1998[	Strong
Catechol-*O*-methyltransferase (COMT)	No	Minor	Yes	SNP in exon 4 (Val^158^Met; rs4680)	rs4680 alters COMT enzyme thermostability, resulting in 40% lower activity in Met allele carriers at body temperature [Chen et al., 2004[	Strong
Serotonin transporter (SLC6A4) linked polymorphic region (5HTTLPR)	Yes	No	No	43-bp VNTR and SNP (rs25531) in promoter	Threefold reduction in transporter activity in the short (S) relative to the long (L) allele of the VNTR [Heils et al., 1996; Lesch et al., 1996[. SNP rs25531, immediately upstream of the VNTR [Kraft et al., 2005; Wendland et al., 2006[ reduces expression of L allele carriers back to levels equivalent to S carriers [Hu et al., 2006[	Weak

**FIG 1:**
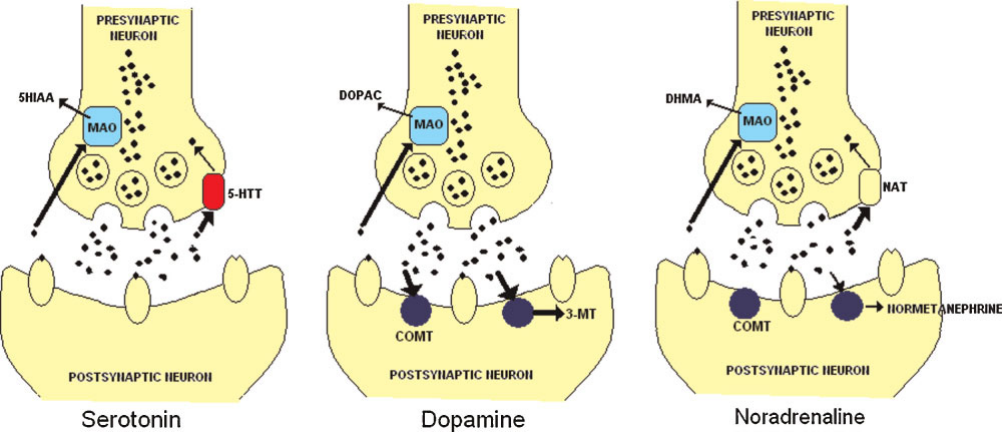
Schematic diagram of major routes of inactivation of the monoamine neurotransmitters serotonin, dopamine, and NA in prefrontal cortical synapses. The enzyme monoamine oxidase, located in the mitochondrial cell wall degrades 5-HT, DA, and NA into the compounds 5HIAA, DOPAC, and DHMA. A second enzyme, catechol-*O*-methyltransferase (COMT) degrades dopamine and, to a lesser extent, NA, forming the products 3-methoxytyramine (3-MT) and normetanephrine. Transporter proteins located in the cell membrane allow reuptake of serotonin and NA into the presynaptic neuron, but dopamine transporters are predominantly located extrasynaptically in primate prefrontal cortex [Lewis et al., 2001[, increasing the importance of enzymatic degradation of DA.

Despite a number of studies in these and other genes, few associations between genetic polymorphisms and cognition have been convincingly replicated. In the largest candidate gene study of cognition to date, we previously reported small but significant sex-specific effects of Val^158^Met on cognitive performance at ages 8 and 10 in a population-based sample of more than 5,000 children from the Avon Longitudinal Study of Parents and Children (ALSPAC) [Barnett et al., 2007[. Among boys, but not girls, Met allele carriers showed better verbal IQ and verbal response inhibition at age 8, and better working memory at age 10. Here we report the effects in the same sample of functional polymorphisms in *MAOA* and *5HTTLPR*. We also assess evidence for epistasis between the *MAOA*, *5HTTLPR*, and *COMT* polymorphisms, in an attempt to broaden the candidate gene approach to include assessment of functionally interactive gene systems, here represented by the overlapping effects of these SNPs on synaptic neurotransmitter availability. We hypothesized that genes that exclusively affect serotonergic function (*5HTTLPR*) would show little effect on these prefrontally driven cognitive tasks, but that main genetic effects, and potential epistasis, would be demonstrated for variants affecting dopaminergic or noradrenergic availability (*COMT*, *MAOA*; see Table I and Fig. 1).

## MATERIALS AND METHODS

### Sample

ALSPAC is a general population cohort based in southwest England. The cohort comprised 14,062 live births from 14,541 enrolled pregnancies which were due to give birth between 01 March 1991 and 31 December 1992 [Golding et al., 2001[. This study is based on cognitive assessments completed at ages 8 and 10. To increase sample homogeneity, only children of Caucasian ethnicity (95% of the cohort) were included in these analyses. Parents gave informed consent at enrolment and ethical approval was obtained from the ALSPAC and Local Research Ethics Committees.

### Cognitive Assessments

As in our previous study [Barnett et al., 2007[, we examined associations between genotype and prefrontal-dependent tests of cognition completed at ages 8 (mean 8 years 8 months, SD 3.1 months) and 10 (mean 10 years 8 months, SD 3.0 months). At age 8, verbal and performance IQ was assessed using alternate items from the Wechsler Intelligence Scale for Children (WISC) 3rd UK edition [Wechsler et al., 1992[ and verbal inhibition was assessed with the Opposite Worlds task from the Test of Everyday Attention for Children battery [Manly et al., 2001[, which required the child to read aloud a string of the digits 1 and 2 and responded in the “opposite” manner (i.e., saying “one” for the digit 2). Working memory was assessed at age 10 using the Count Span task [Case et al., 1982[, which required the child to count out loud the number of red dots presented on a screen. After multiple screens the child was asked how many dots were on each screen within that set, and the number of trials correct (maximum 42) was reported.

### Genotyping

DNA, obtained from blood and mouthwash samples, was extracted and processed [Jones et al., 2000[, and *COMT* Val/Met [Barnett et al., 2007[, and *5HTTLPR* [Araya et al., 2009[ genotyping were conducted as previously described. For *MAOA*, because of the high GC content around the VNTR, amplifications were performed using Invitrogen's PlatinumTaq and PCRX Enhancer System kits, according to the manufacturer's protocol (Invitrogen, Carlsbad, CA), with 5 µM of each primer and 25 mM dNTPs in a total reaction volume of 25 µl. Amplifications were performed on a Perkin Elmer 9700 thermocycler (Applied Biosystems, Foster City, CA) with one cycle at 96°C for 10 min followed by 30 cycles of 94°C for 15 sec, 60°C for 15 sec, 72°C for 30 sec, and a final 3-min extension at 72°C. The forward primer was labeled with the fluorescent dye 6-FAM, and amplicons were visualized on an ABI 3100 capillary sequencer. Allele sizes (allele 2, 233 bp; allele 3, 263 bp; allele 3.5, 278 bp; allele 4, 293 bp; and allele 5, 323 bp) were determined using Genotyper 2.5 (Applied Biosystems). *MAOA* 3, 3.5, and 4-repeat allele frequencies in girls were consistent with Hardy–Weinberg equilibrium (χ^2^ = 1.27, DF 3, *P* = 0.74).

### Analysis

Individuals were classified into high, medium, or low activity groups with respect to the functional effects of the polymorphism in each of the three genes. In each case, “high activity” groups comprised the alleles leading to fastest clearance of the synaptic neurotransmitter and “low activity” the alleles leading to slowest clearance. For *5HTTLPR*, low activity individuals were those with SS, SL_G_, or L_G_L_G_ genotypes, medium activity were SL_A_ or L_G_L_A_ genotypes, and L_A_L_A_ genotypes were denoted high activity [Hu et al., 2006[ since this genotype results in increased serotonin transporter protein and hence faster 5-HT uptake [Lesch et al., 1996[. For *COMT*, Met/Met individuals were considered low, Val/Met individuals medium, and Val/Val individuals high activity, because Val allele carriers show higher COMT enzyme activity, and hence faster degradation of catecholamines at body temperature [Lachman et al., 1996; Chen et al., 2004[. For *MAOA* we excluded genotypes involving the rare 2- or 5-repeat alleles, whose functional effects are not yet clear [Sabol et al., 1998; Deckert et al., 1999[. Since *MAOA* is located on the X chromosome, boys carry only one allele. Boys with the 3-repeat allele were denoted low *MAOA* activity and those with 3.5- or 4-repeat alleles as high activity, since they show higher transcriptional efficiency and hence result in higher levels of synaptic *MAOA* [Sabol et al., 1998; Deckert et al., 1999[. For girls, 3/3 homozygotes were denoted low activity, 3/3.5 or 3/4 heterozygotes medium activity, and genotypes comprising two copies of 3.5- or 4-repeat alleles were denoted high activity.

Main effects of genes on cognition were assessed by comparing cognitive scores between high, medium, and low activity groups using one-way ANOVA for the *5HTTLPR*, and for *MAOA* in girls only. For boys, we compared cognitive scores in high versus low activity groups using *t*-tests. Main effects of *COMT* in this sample have been previously reported [Barnett et al., 2007[ and hence were not assessed here.

Two possible sources of epistatic effects on cognition were considered: a serotonergic interaction between *5HTTLPR* and *MAOA* genotypes, and a catecholaminergic one between *COMT* and *MAOA* genotypes. We assessed evidence for these interactions using general linear models with each cognitive measure as an outcome variables predicted by two genetic main effects and a gene–gene interaction term. As before, all genotypes were coded as low, medium, or high activity. These analyses were conducted separately in boys and girls because *MAOA* is located on the X chromosome, and because *COMT* is known to have sexually dimorphic effects on a range of functional and neuropsychiatric phenotypes [Harrison and Tunbridge, 2008[, including cognitive function in this sample [Barnett et al., 2007[.

## RESULTS

### Data Availability

The number of children included in each analysis varied by the availability of cognitive data and genotypes: cognitive data were available on around two-thirds of those for whom DNA is available. As previously reported [Araya et al., 2009[, after stringent quality control measures, fewer children were available for inclusion in analyses involving *5HTTLPR* genotype. There were no cognitive differences between children for whom *5HTTLPR*, *MAOA*, or *COMT* genotypes were or were not available (all *P* > 0.05). Allele frequencies were, for *5HTTLPR* alleles S = 41.4%, L_A_ = 51.5%, L_G_ = 7.1%, and for *MAOA*, 2-repeat = 0.2%, 3-repeat = 34.0%, 3.5-repeat = 2.0%, 4-repeat = 62.4%, 5-repeat = 1.5%. *COMT* allele frequencies, as previously reported, were Val = 48.6%, Met = 51.4%. The number of children considered as low, medium, and high activity genotypes for whom at least one cognitive measure was available are shown in Table II.

**TABLE II:** Sample Sizes of High, Medium, and Low Activity MAOA, 5HTTLPR, and COMT Genotype Groups in ALSPAC Cognitive Study

	High activity		Medium activity		Low activity	
Total (n)	Genotypes	n	Genotypes	n	Genotypes	n
MAOA girls						
2,984	3.5/3.5; 3.5/4; 4/4	1,245	3/3.5; 3/4	1,380	3/3	359
MAOA boys						
3,066	3.5; 4	2,037	NA	0	3	1,029
5HTTLPR						
4,579	L_A_L_A_	1,153	SL_A_; L_G_L_A_	2,346	SS; SL_G_; L_G_L_G_	1,080
COMT						
5,909	Val/Val	1,396	Val/Met	2,921	Met/Met	1,592

In each case “high activity” refers to the allele resulting in faster clearance of synaptic neurotransmitter.

### Main Effects of *MAOA*

Effects of *MAOA* on cognitive measures were assessed separately in boys and girls. *t*-Tests between high (minimum n = 1,685) and low (minimum n = 854) activity *MAOA* groups in boys showed no effects on any cognitive measure. Similarly in girls, one-way ANOVA between low, medium, and high activity *MAOA* groups (minimum sample sizes, respectively, 290, 1,170, 1,024) revealed no cognitive differences (Table III).

**TABLE III:** Main Effects of MAOA and 5HTTLPR Genotype on Cognitive Function in ALSPAC

Measure	Total (n)	Low	Medium	High	*F*	DF	*P*-value
5HTTLPR							
Verbal IQ	4,174	107.5 (16.6)	107.8 (16.7)	108.5 (16.2)	1.20	2,4171	0.30
Performance IQ	3,882	100.4 (16.6)	100.4 (16.8)	100.5 (16.1)	0.05	2,3879	0.95
Verbal inhibition	4,109	0.244 (0.026)	0.244 (0.025)	0.245 (0.025)	0.55	2,4106	0.58
Working memory	3,880	18.7 (7.49)	18.5 (7.75)	19.2 (7.58)	3.13	2,3877	0.04
MAOA boys							
Verbal IQ	2,717	108.2 (17.2)	—	108.1 (17.2)	0	1,2715	0.99
Performance IQ	2,555	99.6 (17.0)	—	99.5 (17.2)	0.01	1,2553	0.93
Verbal inhibition	2,678	0.242 (0.025)	—	0.242 (0.025)	0.38	1,2676	0.54
Working memory	2,537	18.4 (8.06)	—	18.3 (7.87)	0.25	1,2535	0.62
MAOA girls							
Verbal IQ	2,673	106.8 (15.4)	107.3 (15.8)	106.9 (15.9)	0.19	2,2670	0.82
Performance IQ	2,484	100.9 (16.1)	100.1 (16.2)	101.1 (16.5)	1.17	2,2481	0.31
Verbal inhibition	2,643	0.247 (0.025)	0.246 (0.025)	0.246 (0.025)	0.25	2,2640	0.78
Working memory	2,565	18.8 (7.62)	18.8 (7.13)	18.8 (7.55)	0.04	2,2562	0.96

### Main Effects of *5HTTLPR*

Groups defined by low, medium, and high serotonin transporter promoter activity were compared on all cognitive measures using one-way ANOVA. There were no differences between groups on measures of IQ or verbal inhibition (see Table III). Nominal differences were seen between groups on working memory score (*F* = 3.13, DF 2,3877, *P* = 0.04, uncorrected) such that better performance was seen in the high than medium activity group (Tukey's post hoc *P* = 0.03). One-way ANOVA revealed no cognitive differences between the three genotypes (SS, SL_G_, and L_G_L_G_) within the low activity group.

### Gene × Gene Interactions

**FIG 2:**
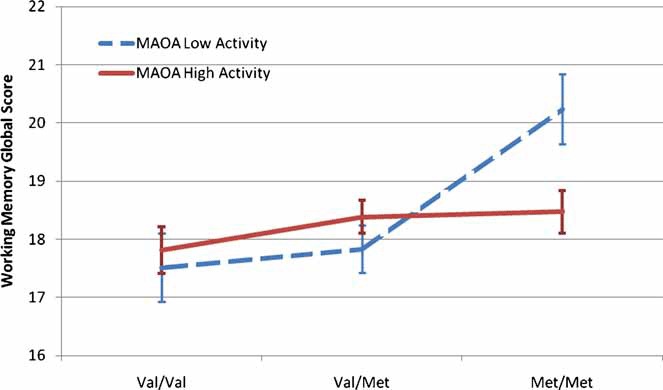
Working memory global score (mean, SE) by COMT and MAOA genotype in 2,324 boys at age 10 years. COMT × MAOA interaction term: *F* = 4.17; DF 2,2324, *P* = 0.016.

Models comprising main effects of *COMT* genotype and *MAOA* genotype, and a *COMT* × *MAOA* interaction term, were fitted separately for girls and boys. In boys, there was evidence of interaction between *COMT* and *MAOA* only for working memory score (model fit *F* = 3.54, DF 5,2324, *P* = 0.003; main effect of COMT *F* = 6.89, DF 1,2324, *P* = 0.001; main effect of MAOA *F* = 0.68, DF 1,2324, *P* = 0.41; COMT × MAOA interaction term *F* = 4.17; DF 2,2324, *P* = 0.016; see Fig. 2). Consistent with our previous report [Barnett et al., 2007[, these models showed main effects of COMT genotype on verbal IQ, verbal inhibition, and working memory, in all cases such that Met alleles were advantageous. In contrast, in girls there were no significant main effects nor interaction terms for any cognitive outcome.

Models comprising main effects of *MAOA* and *5HTTLPR*, and a *MAOA* × *5HTTLPR* interaction term, did not show significant fit for any cognitive measure in either boys or girls.

## DISCUSSION

In a large, population-based sample, we assessed the cognitive effects of functional polymorphisms in three genes which have predictable and partially overlapping effects on serotonin, DA, and NA availability in the PFC. We found no evidence for effects of serotonin availability on measures of IQ, verbal inhibition, or working memory, and no evidence for cognitive effects of interactions between the *5HTTLPR* and *MAOA*. Contrary to our hypotheses, we found no evidence for main effects of *MAOA* variation on cognitive functions, but there were apparent interactions between the *COMT* and *MAOA* genes in their effects on working memory in boys. Here we consider possible explanations for this pattern of limited and sex-specific cognitive genetic effects.

The lack of serotonergic effects on prefrontal cognition reported here fit with the majority of previous evidence, where serotonergic manipulations, including variation in *5HTTLPR* have been previously reported to affect aspects of decision-making and attentional control [Beevers et al., 2007; Homberg et al., 2008; Fox et al., 2009; Roiser et al., 2009[ but not IQ or response inhibition [Fallgatter et al., 1999; Clark et al., 2005; Payton et al., 2005; da Rocha et al., 2008[. Unlike previous studies, the present sample was sufficiently large to detect even very small associations between genotype and cognition (93% power to detect the effect of a locus explaining just 0.2% of variance in the sample, given n = 6,000, alpha = 0.05, and assuming no dominance). We can therefore definitively say that there is no evidence for cognitive effects of *5HTTLPR* on these measures of working memory, response inhibition, or IQ in healthy children. It remains plausible, however, that genetic effects on cognition are seen in circumstances in which the serotonergic *status quo* has been disrupted, for example, by stress, tryptophan depletion [Roiser et al., 2007; Firk and Markus, 2009[ or in neuropsychiatric disorders [Beevers et al., 2007; da Rocha et al., 2008[. Some apparently conflicting results may therefore have arisen because cognitive differences between genotypes appear exaggerated in circumstances of serotonergic perturbation, where normal mechanisms of compensation for genetic variation are disturbed.

It is notable that no genetic associations were observed on the Opposite Worlds verbal inhibition task, other than the previously reported main effects of *COMT* genotype [Barnett et al., 2007[. While previous reports of associations between response inhibition tasks and *5HTTLPR* have been negative [Fallgatter et al., 1999; Clark et al., 2005[, a recent neuroimaging study found both main effects of *5HTTLPR* and *MAOA* polymorphisms, and interactions between the two on anterior cingulate cortex activity elicited by a Go/NoGo response inhibition task in healthy volunteers [Passamonti et al., 2008[. The lack of interaction between *5HTTLPR* and *MAOA* in our study may in part reflect the fact that while MAOA is more abundant in catecholaminergic neurons, MAOB is more abundant in serotonergic neurons [Levitt et al., 1982; Saura et al., 1992[.

Although the lack of cognitive effects of *5HTTLPR* were predictable given the cognitive domains assessed, it is somewhat surprising that no main effect was detected for *MAOA*. The negative main effects of *MAOA* reported here are in contrast to previous reports of IQ effects in a sample of healthy Chinese females [Yu et al., 2005[, two samples of autistic children from Israel [Yirmiya et al., 2002[ and Canada [Cohen et al., 2003[, and a sample of boys with ADHD from Israel, where the *MAOA* low activity allele was associated with better performance on a continuous performance test [Manor et al., 2002[. There are a number of differences between this and the previous, positive reports including ethnicity, sex distribution, and developmental stage, which might conceivably affect the genetic effect on cognition among samples. For example, *MAOA*'s effects may be limited to one particular developmental stage, or in previous reports may have been due to a second locus that shows differential linkage disequilibrium between ethnic groups. The sex-specific analyses presented here reduced statistical power through halving the sample size (reducing to around 69% the power to detect an association explaining 0.2% of cognitive variance). Nonetheless, this sample remains the largest so far examined with respect to *MAOA* and cognition, and the complete lack of difference in cognitive score between genotypes in either sex is again a decisive negative result.

In contrast to the absence of main effects of *MAOA*, there was a modest but significant interactive effect of *MAOA* genotype and *COMT* genotype on working memory performance in boys but not girls. With few exceptions, cognitive genetic studies have thus far had inadequately sized samples to realistically assess possible gene–gene (epistatic) interactions. The *COMT* × *MAOA* interaction observed here for working memory is plausible given the dependence of working memory on prefrontal dopaminergic availability. That no similar effect was found for IQ is surprising: *COMT* genotype has a strong, male-specific effect on verbal IQ in this sample [Barnett et al., 2007[, and previous evidence has supported an effect of *MAOA* on IQ but not measures of executive function [Yu et al., 2005[.

Separate analyses were carried out for boys and girls and for multiple cognitive phenotypes. Since scores on the four cognitive measures were correlated, strict correction for multiple comparisons using, for example, a Bonferroni correction, was not suitable. Nonetheless, while the large sample used in the study reduces the chances of Type II error, the possibility of Type I error remains present.

While both tasks that have a high working memory content [Owen, 1997; Owen et al., 2005[ and those that load highly on general intelligence [Duncan et al., 2000[ typically activate a range of lateral frontal cortical areas, there are nonetheless task-specific differences in cognitive demands. Plausible explanations for the association between catecholaminergic genes and working memory, but not IQ, therefore involve the specific neural underpinnings of the different tasks, and the complexity of gene expression within different regions of the brain. While *MAOA* has a widespread distribution in the brain [Grimsby et al., 1990[, it has recently been suggested that frontal cortical *MAOA* expression may be predominantly determined by genetic and epigenetic factors other than this VNTR, including a currently unknown locus with which it shows strong linkage disequilibrium [Balciuniene et al., 2002; Pinsonneault et al., 2006[. Similarly, while *COMT* expression appears greater in PFC than other regions [Matsumoto et al., 2003[, recent studies have shown that the expression of gene products of *COMT* is complex, with tissue-specific monoallelic expression [Gimelbrant et al., 2007[, multiple mRNA variants expressed at different levels across the brain [Tunbridge et al., 2007a[, and differences in both mRNA and protein expression across developmental stages [Tunbridge et al., 2007a,b[. It is also important to note that variation at any one locus in a gene may not act alone in exerting cognitive effects. For example, we have recently reported the effects of additional *COMT* SNPs that act in concert with the Val^158^Met polymorphism in influencing cognitive function in this sample [Barnett et al., 2009[. While the inclusion of additional SNPs further reduces group sizes, and hence statistical power, the more accurate functional characterization gained by haplotype analysis may sometimes offset this loss of power. In this study, re-analysis using *COMT* haplotype groups produced no meaningful change in results from those seen using just the Val^158^Met genotype (see Supplementary Fig. 1 for the effects of *COMT* haplotype characterization of the *COMT* × *MAOA* interaction on boys' working memory).

The gene–gene interaction found here suggests that *MAOA* genotype affects working memory only in the context of high prefrontal catecholamine availability. There is, of course, a distinction between demonstrating a statistical interaction, and understanding the biological mechanism of interaction between two genes [Clayton and McKeigue, 2001[. Further studies will clearly be needed both to replicate this effect on working memory and to clarify the biological pathway of any such interaction. While prefrontal dopaminergic function is one obvious candidate, there may be other means by which *COMT* and/or *MAOA* expression affect cognitive function. One such possibility is NA, which, like DA, is catabolized to some extent by both *COMT* and *MAOA*. At present, dopaminergic pathways remain the more obvious candidate, because COMT appears to play a relatively minor role in regulating NA in the PFC [Gogos et al., 1998; Tunbridge et al., 2004[.

The sex-specific nature of *COMT* × *MAOA* effect on working memory was in accordance with our previous study [Barnett et al., 2007[, which showed that *COMT* genotype affected a wide range of cognitive functions in boys but had no effect in girls. There is additional wide-ranging evidence to suggest that the effects of COMT may be different between the sexes [Harrison and Tunbridge, 2008[, for example, DA levels in the frontal cortex are affected only in male *COMT* knockout mice, which show sex-specific behavioral phenotypes [Gogos et al., 1998[, and there are sex differences in both the expression and effects of *COMT* in human PFC [Chen et al., 2004[. Studies in model organisms, where our understanding of genetic variation is further advanced than in humans, confirm the existence of a wide range of sexually dimorphic genetic effects on normal variation in traits such as lifespan, obesity, and skeletal form [Nuzhdin et al., 1997; Farber and Medrano, 2007[. The same appears true in humans where there are known sex-specific effects on reproductive, physiological, and disease traits, probably resulting from differential gene regulation in males and females [Ober et al., 2008[. The likely explanation for sex-specific associations with *COMT* are the bilateral relationships between *COMT* and estrogen-related compounds: estrogens mediate *COMT* expression [Xie et al., 1999[, and *COMT* metabolizes catechol estrogens, a process regulated by Val^158^Met variation [Worda et al., 2003[. In addition to these issues with *COMT*, the location of the MAOA gene on the X chromosome complicates association studies, with the result that little is known about the relative expression and effects of *MAOA* in the two sexes.

In conclusion, we present evidence that functional polymorphisms in *COMT* and *MAOA*, two genes involved in the functional deactivation of DA and other neurotransmitters, show interactive effects on working memory performance in normal children from a large, homogeneous, and representative sample. While neither gene has been unquestionably linked to psychiatric disorder, normal variation in brain function is likely to be partly determined by genetic variation in neurotransmitter pathways. Understanding genetic effects on normal brain function is a necessary stage in understanding the basis of abnormal function, but large samples are required to tease out relatively subtle effects on cognitive development at the population level.
